# The extremely divergent maternally- and paternally-transmitted mitochondrial genomes are co-expressed in somatic tissues of two freshwater mussel species with doubly uniparental inheritance of mtDNA

**DOI:** 10.1371/journal.pone.0183529

**Published:** 2017-08-17

**Authors:** Sophie Breton, Karim Bouvet, Gabrielle Auclair, Stéphanie Ghazal, Bernard E. Sietman, Nathan Johnson, Stefano Bettinazzi, Donald T. Stewart, Davide Guerra

**Affiliations:** 1 Department of Biological Sciences, Université de Montréal, Montréal, Québec, Canada; 2 Minnesota Department of Natural Resources, Division of Ecological and Water Resources, Lake City, Minnesota, United States of America; 3 U. S. Geological Survey, Wetland and Aquatic Research Center, Gainesville, Florida, United States of America; 4 Department of Biology, Acadia University, Wolfville, Nova Scotia, Canada; University of Parma, ITALY

## Abstract

Freshwater mussel species with doubly uniparental inheritance (DUI) of mtDNA are unique because they are naturally heteroplasmic for two extremely divergent mtDNAs with ~50% amino acid differences for protein-coding genes. The paternally-transmitted mtDNA (or M mtDNA) clearly functions in sperm in these species, but it is still unknown whether it is transcribed when present in male or female soma. In the present study, we used PCR and RT-PCR to detect the presence and expression of the M mtDNA in male and female somatic and gonadal tissues of the freshwater mussel species *Venustaconcha ellipsiformis* and *Utterbackia peninsularis* (Unionidae). This is the first study demonstrating that the M mtDNA is transcribed not only in male gonads, but also in male and female soma in freshwater mussels with DUI. Because of the potentially deleterious nature of heteroplasmy, we suggest the existence of different mechanisms in DUI species to deal with this possibly harmful situation, such as silencing mechanisms for the M mtDNA at the transcriptional, post-transcriptional and/or post-translational levels. These hypotheses will necessitate additional studies in distantly-related DUI species that could possess different mechanisms of action to deal with heteroplasmy.

## Introduction

The mitochondrial genome (mtDNA) is strictly maternally inherited in animals, and is maintained in a homoplasmic state (as opposed to heteroplasmy), in which all mtDNA copies are typically genetically identical in each cell [1]. This homoplasmic state prevents potentially harmful genomic conflicts and promotes co-evolution of interacting mitochondrial and nuclear genes for optimal mitochondrial functions [2,3]. Only one exception to the rule of strictly maternal mtDNA inheritance is known in animals. Doubly Uniparental Inheritance (DUI) of mtDNA is characterized by two mitochondrial lineages with extreme DNA divergence (~20–40%): one transmitted through females (F) and the other through males (M) [4–8]. DUI represents a unique, naturally occurring heteroplasmic system, enabling the analysis of the cellular and molecular consequences of the presence of two highly divergent mitochondrial lineages that coexist in the same nuclear background.

So far, the DUI system has been shown to occur in approximately 100 bivalve species belonging to the marine bivalve orders Mytiloida, Veneroida and Nuculanoida, and the freshwater bivalve order Unionoida [9]. In most of the marine mussels *Mytilus* spp. and marine clam *Ruditapes philippinarum* studied, fertilization experiments performed using sperm stained with the mitochondrial dye MitoTracker® Green FM (Molecular Probes®, Eugene, OR, USA) showed that the sperm-transmitted mitochondria clustered together during several of the first cell division cycles and ended up in primordial germ cells in embryos that would become males, whereas the paternally derived mitochondria dispersed and rapidly disappeared in embryos that would become females [10–13]. The simplified scenario from *Mytilus* spp. and *R*. *philippinarum* is that only males are heteroplasmic, with the F mtDNA found in all somatic tissues and the M mtDNA only in the germ line. However, many exceptions to this “simplified mitochondrial portrait” in DUI species have been reported in the recent years (reviewed in [6]). For example, in *Mytilus* spp. both genomes have been found in somatic tissues of both sexes, with the M mtDNA usually present at extremely low concentrations in somatic tissues compared to the F mtDNA, and the presence of the M genome, also in extremely small quantities, has been reported in eggs [6]. By contrast, male somatic tissues can contain high quantities of M mtDNA in *R*. *philippinarum*, even more than the F mtDNA, whereas heteroplasmic females are rather uncommon in this species [14]. Studies in *Mytilus* spp. and *R*. *philippinarum* have shown that spawned sperm, purified in Percoll^TM^, contained only M mtDNA; obviously, such sperm should only express the M mtDNA (reviewed in [6]). As emphasised by Dalziel and Stewart [15], Zouros [6] and Milani et al. [16], an important question in DUI is whether the M genome is indeed expressed. It is known that mitochondrial heteroplasmy can be deleterious in biological systems other than DUI (e.g., [17]), and lead to degenerative diseases in humans (e.g., [18]), hence the presence of two highly divergent mtDNAs in somatic (or gonadal) tissues of DUI species raised the question as to whether both genomes are indeed producing proteins.

At the transcriptional level, it seems that both F and M mtDNAs can be expressed when present in somatic tissues, although little data are currently available. In *Mytilus edulis* for example, M mtDNA transcripts have been detected, using reverse transcription polymerase chain reaction (RT-PCR), not only in male gonads but also in 50% of the male somatic tissues tested and in only one female somatic tissue sample (8% of the somatic tissues tested) [15]. However, the authors did not know *a priori* if their somatic tissue samples were heteroplasmic or not, meaning that it was impossible to conclude if the M mtDNA was always expressed or not when present in somatic tissues. This idea has been tested in a more recent study in *Mytilus galloprovincialis*, using quantitative PCR (qPCR), quantitative reverse transcription PCR (RT-qPCR) and *in situ* hybridization with probes specific to M and F mtDNAs [19]. The authors concluded that female and male somatic tissues and female gonads had low M mtDNA levels, but that it was not expressed, and therefore, they proposed different systems for M and F mtDNA tissue-specific transcriptional regulation with F mtDNA being functional in somatic tissues and female gonads, while M mtDNA in spermatogenetic cells only [19]. This contrasts somewhat with the results obtained for *M*. *edulis* but also with the results recently obtained for the marine clam *R*. *philippinarum*, in which M mtDNA expression has been detected in all male heteroplasmic somatic tissue samples examined using RT-qPCR [16]. In this last study, female somatic tissues were not examined, but as mentioned above, heteroplasmic females are rarely found in this species [14]. Worldwide, three studies using different techniques to detect M mtDNA expression in female and male bivalve somatic tissues have produced different results, and thus it is still unclear whether a similar pattern of M mtDNA expression exists in all DUI species or if evolutionary expression changes have occurred in the various bivalve groups/species.

No detailed study of the expression of the M mtDNA in somatic tissues of unionoid freshwater bivalves and nuculanoid marine bivalves has been performed to date. In unionoids, the M mtDNA has been detected in male somatic tissues of *Unio crassus* but not in females, which lead the authors to propose a new method of sex identification in this endangered species using minimally invasive tissue sampling [20]. Also, protein-based studies showed evidence of weak expression of the M mtDNA in male somatic tissues of *Venustaconcha ellipsiformis* but not in female somatic tissues [21], and for expression of the M mtDNA in both male and female gonads [22]. However, no study of RNA levels has been done yet. In the present study, we used PCR and RT-PCR to detect the presence and possible expression, respectively, of the M mtDNA in male and female gonadal and somatic tissues of two freshwater mussel species, *Venustaconcha ellipsiformis* (Unionidae, Tribe Lampsilini) and *Utterbackia peninsularis* (Unionidae, Tribe Anodontini). Based on what was found in other DUI species, we hypothesized that (1) although both F and M mtDNAs can be present in male and female somatic and gonadal tissues, (2) they are co-expressed only or mainly in male somatic and gonadal tissues. We aimed to elucidate whether sex- and/or tissue-specific transcriptional regulation for M mtDNA exist in freshwater species with DUI.

## Materials and methods

### Specimen collection and sample preparation

Sixteen adult specimens of *Venustaconcha ellipsiformis* and fifteen adult specimens of *Utterbackia peninsularis* were collected in July 2014 from Straight River (Minnesota, USA; Lat 44.006509, Long -93.290899) and Suwannee River (Florida, USA; Lat 29.58684, Long -82.94095), respectively. *U*. *peninsularis* is not a protected species at state or federal or international levels and no special permissions were needed for its collection and study. *V*. *ellipsiformis* is state threatened, but the specimens were collected under authority of the Minnesota DNR and no permit was required (also the population in the Straight River is healthy).

All mussels were considered reproductively mature with total lengths >

40mm (see S1 Fig). Mussels were shipped alive to the Université de Montréal. Each individual was sexed by microscopic examination of gonad smears, and only unambiguously sexed specimens, i.e., those with swimming sperm or mature eggs, were kept for further analyses (12 for *V*. *ellipsiformis* and 13 for *U*. *peninsularis*). Samples were obtained from somatic (gill, foot, mantle and adductor muscle) and gonadal tissues, using precautions to avoid contamination between male and female tissues. These precautions included (1) using fresh cleaned or sterilized glassware, plastic ware, pestles and scalpels for each individual, and (2) using fresh, sterile scalpels to dissect each tissue to avoid cross-contamination. All tissues (two replicates per sample) were frozen at -80°C until further analyses.

### Tissular distribution of the M mitochondrial genome

A total of 116 DNA samples (58 for each species) was extracted from somatic and gonadal tissues, with the MasterPure Complete DNA and RNA Purification Kit (Epicentre). The quality and quantity of extracted DNA were examined via electrophoresis on 1% agarose gels and a BioDrop μLITE spectrophotometer. Different pairs of M-specific primers were used, i.e, either already published (e.g., HCO700dy2 and UNIOCOII2; [23]) or *de novo* designed with Primer3Plus (at primer3plus.com; [24]) for both species on the basis of complete M mitochondrial sequences (GenBank accession numbers FJ809752.1 and HM856635.1; [25]) (see Table 1). For both species, the tissular distribution of the M genome was investigated with primers designed to amplify the male-specific M-*orf* gene [26], the male *cox2*-*cox1* region (see below) and the male *cox1* gene (only for *V*. *ellipsiformis*). Partial sequence amplifications were carried out in 25 μl volumes comprising 2.5 μl 10X Taq buffer, 0.5 μl dNTP mix (10mM), 1.0 μl of each forward and reverse primer, 0.125 μl Taq DNA Polymerase (5U/ml; Bio Basic Inc., Markham, Ontario, Canada), 1 μl of DNA extract (~100 ng/μl), and ddH_2_O up to 25 μl. For M-*orf* and M*cox1*, PCR reactions were performed on a TProfessional Basic Thermocycler with the following conditions: initial denaturation at 95°C for 2 min, followed by 35 cycles of 95°C for 20 sec, 58°C for 30 sec for M-*orf* and 58°C for 1 min for M*cox1*, and 72°C for 20 sec for M-*orf* and for 40 sec for M*cox1*, followed by a final extension step at 72°C for 5 min. To ensure that the correct fragment of mtDNA was amplified, two PCR products for each species, i.e., one for M*cox1* and one for M-*orf*, were purified with the Qiagen QIAquick PCR Purification Kit according to the manufacturer’s protocol. The purified PCR products were sequenced at the Institute for Research in Immunology and Cancer Institute (IRIC, University de Montréal, Montreal, Canada), and the sequences were compared to those available in GenBank (accession numbers FJ809752.1 and HM856635.1; [25]).

**Table 1:** Details of primers used in this study for the amplification of M-specific segments.

Gene target	Species	Primer name	5'-3' sequence
M-*orf*	*V*. *ellipsiformis*	V-M-For-1	ACACAAACCAGACCCAATACAAGT
		V-M-Rev-3	GGGTTTGTTCGCGGTATTGTG
	*U*. *peninsularis*	Utt-morf-for	AAAGAAGCTGTTAAAGAGGCT
		Utt-morf-rev	ATCTTTCGTAATCACCAACTC
*cox2*-*cox1*	*V*. *ellipsiformis &*	HCO700dy2*	TCAGGGTGACCAAAAAAYCA
	*U*. *peninsularis*	UNIOCOII2*	CAGTGGTATTGGAGGTATGAGTA
M*cox1*	*V*. *ellipsiformis*	VeMcox1-FOR3	TCATCCTGGCGGTGTGATTC
		VeMcox1-REV3	CCCCTCCAGACGGATCAAAA
	*U*. *peninsularis*	UpMcox1-F2	TTGGATGTGGACACTCGAGC
		UpMcox1-R2	ACACCGCTGAGAGCAAAGAA
M*cox2e*	*U*. *peninsularis*	Utt-cox2e-for	ATAGACAGAAGCATCAATTGG
		Utt-cox2e-rev	AAGGTTGTATGTCTGGTGG

Note.

*; these primers were designed by [25], all the other primers have been *de novo* designed for the present study.

The primers HCO700dy2 and UNIOCOII2 are called M-specific but they amplify approximately 1.1 kb of *cox2*-*cox1* from F genomes and approximately 1.7 kb from M genomes because of the presence of the M genome-specific 3’ extension of the *cox2* gene (M*cox2e*) in freshwater mussels [23]. Hence, these primers served not only to detect the presence of the M mtDNA but also as an internal control–because all individuals are expected to carry an F mtDNA molecule–against scoring a sample as lacking the M mtDNA when in fact neither the M nor the F could be detected. All resulting PCR products were visualized on 1% (for the bigger amplicons M*cox2*-*cox1* and M*cox1* of 1.1–1.7 kb and ~500 bp, respectively) or 2% (for the smaller amplicon M-*orf* of ~250 bp) agarose gels under UV light with SYBR green dye (Life Technologies).

### Tissular expression of the M mitochondrial genome

Total RNA was extracted from tissues that tested positive for the presence of the M mtDNA with the MasterPure Complete DNA and RNA Purification Kit (Epicentre) and treated with Turbo DNase (AMBION, Austin, TX, USA) following the provided protocols to remove DNA contamination that could be amplified in the RT-PCRs, resulting in false-positive results. The extracted RNA was quantified using a BioDrop μLITE spectrophotometer. cDNA was synthesized with ImProm-II Reverse Transcriptase (Promega) following the manufacturer’s protocol.

For both species, the expression of the M genome was investigated with primers designed to amplify the male-specific M-*orf*, M*cox1* or M*cox2e* (*U*. *peninsularis* only) genes (Table 1). PCR assays were carried out in 25 μl volumes comprising 2.5 μl 10X Taq buffer, 0.5 μl dNTP mix (10mM), 1.0 μl of each forward and reverse primer, 0.125 μl Taq DNA Polymerase (5U/ml; Bio Basic Inc., Markham, Ontario, Canada), 1 μl of cDNA (~500 ng/μl), and ddH_2_O up to 25 μl. Reactions were performed on a TProfessional Basic Thermocycler with the following PCR amplification conditions: initial denaturation at 95°C for 2 min, followed by 35 cycles of 95°C for 20 sec, 58°C for 30 sec for M-*orf* and M*cox2e* and 58°C for 1 min for M*cox1*, and 72°C for 20 sec for M-*orf* and *Mcox2e* and for 40 sec for M*cox1*, followed by a final extension step at 72°C for 5 min. All resulting PCR products were visualized on 1% (for M*cox1*) or 2% (for M-*orf* and M*cox2e*) agarose gels under UV light with SYBR green dye (Life Technologies).

Finally, to control against scoring a sample as showing no M mtDNA expression when in fact neither expression of the M nor the F could be detected, we used the universal primers LCO1490 and HCO2198 for *cox1* [27], which can amplify both F and M genomes in species with DUI [9]. A band of the expected size was detected in all samples.

## Results

### Detection of mitochondrial heteroplasmy

Thirteen animals were analyzed for *U*. *peninsularis* (5 females and 8 males) and twelve animals were analyzed for *V*. *ellipsiformis* (6 females and 6 males). The results of PCR amplifications to detect the presence of M mtDNA are given in Table 2 (note: the F genome was detected in all samples). Of 58 *V*. *ellipsiformis* tissues analyzed, 30 out of 58 (51.7%) produced an M-specific band. In turn, 5 out of 28 (17.9%) female tissues were found to be heteroplasmic: M mtDNA was identified only in female mantle and adductor muscle tissues, whereas gonad, gill, and foot tissues never showed a detectable M band. Male gonad and foot tissues were always heteroplasmic (gonad tissue is a mixture of gametes and supportive somatic tissues), and the majority of the other male tissues produced an M amplicon (83.3%). In *U*. *peninsularis*, M was amplified in 41 out of 58 (70.7%) of the tissues analyzed. A majority of female tissues were found to be heteroplasmic (72.7%), even female gonad tissues (4 out of 5). In males, 69.4% of tissues (25 out of 36) were found to be heteroplasmic and as expected, male gonads were always heteroplasmic.

**Table 2:** Results of the PCR experiments on all samples tested.

		Tissues					
Species	Specimen	Gonad	Gill	Foot	Mantle	Adductor	heteroplasmic tissues
*V*. *ellipsiformis*	♀2	-				*	1/4
	♀3				*	*	2/5
	♀4				*		1/5
	♀5					*	1/5
	♀6					-	0/4
	♀12						0/5
	♂1	*	*	*		*	4/5
	♂7	*	*	*	*	*	5/5
	♂8	*	*	*	*		4/5
	♂9	*	*	*	*	*	5/5
	♂10	*		*		*	3/5
	♂11	*		*	*	*	4/5
*U*. *peninsularis*	♀2			*		-	1/4
	♀4	*	*			-	2/4
	♀7	*	*	*	*	*	5/5
	♀14	*	*	*	*	*	5/5
	♀19	*	*	*		-	3/4
	♂1	*		*			2/5
	♂3	*		*		-	2/4
	♂5	*				-	1/4
	♂8	*	*	*	*	-	4/4
	♂11	*	*	*	*	*	5/5
	♂15	*	*	*	*	*	5/5
	♂17	*	*	*	*		4/5
	♂18	*			*	-	2/4
*V*. *ellipsiformis*	all ♀	0/5	0/6	0/6	2/6	3/5	5/28
	all ♂	6/6	4/6	6/6	4/6	5/6	25/30
*U*. *peninsularis*	all ♀	4/5	4/5	4/5	2/5	2/2	16/22
	all ♂	8/8	4/8	6/8	5/8	2/4	25/36

Note. Sex of specimens is specified with ♀ for females and ♂ for males. In the last column and at the bottom of the table, a summary of the results is given respectively for each specimen and sex in the form “number of heteroplasmic tissues / total number of tissues”. Empty cells in the “Tissues” columns indicate negative PCR results for M targets. Symbols used

*, positive amplification of M-*orf* and M*cox2-cox1* and/or M*cox1*; -, sample not available.

### Detection of M-specific mitochondrial transcription

The 30 and 41 heteroplasmic tissues of *V*. *ellipsiformis* and *U*. *peninsularis*, respectively, were analysed to detect transcription of M mtDNA (results are shown in Table 3). As expected, M mtDNA expression was detected in all male gonad samples. For *V*. *ellipsiformis*, 1 out of 5 (20%) female heteroplasmic tissues was found to express the M mtDNA (an adductor muscle), whereas 17 out of 19 (89.5%) male somatic heteroplasmic tissues expressed the M mtDNA. For *U*. *peninsularis*, 1 out of 15 (6.7%) female heteroplasmic tissues expressed the M mtDNA (a foot), whereas 11 out of 17 (64.7%) male somatic heteroplasmic tissues were found to express the M mtDNA.

**Table 3:** Results of the PCR experiments performed on total cDNA from all somatic heteroplasmic tissues of *V*. *ellipsiformis* and *U*. *peninsularis*.

		Tissues					
Species	Specimen	Gonad	Gill	Foot	Mantle	Adductor	M-transcribing heteroplasmic tissues
*V*. *ellipsiformis*	♀2	-				*	1/1
	♀3						0/2
	♀4						0/1
	♀5						0/1
	♀6					-	na
	♀12						na
	♂1	*	*	*		*	4/4
	♂7	*	*	*	*	*	5/5
	♂8	*		*	*		3/4
	♂9	*	*	*		*	4/5
	♂10	*		*		*	3/3
	♂11	*		*	*	*	4/4
*U*. *peninsularis*	♀2			*		-	1/1
	♀4					-	0/2
	♀7						0/5
	♀14						0/5
	♀19	-				-	0/2
	♂1	*		*			2/2
	♂3	*		*		-	2/2
	♂5	*				-	1/1
	♂8	*	*	*		-	3/4
	♂11	*	*	*			3/5
	♂15	*		*		*	3/5
	♂17	*	*	*			3/4
	♂18	*			*	-	2/2
*V*. *ellipsiformis*	all ♀	na	na	na	0/2	1/3	1/5
	all ♂	6/6	3/4	6/6	3/4	5/5	23/25
*U*. *peninsularis*	all ♀	0/3	0/4	1/4	0/2	0/2	1/15
	all ♂	8/8	3/4	6/6	1/5	1/2	19/25

Note. Sex of specimens is specified with ♀ for females and ♂ for males. In the last column and at the bottom of the table, a summary of the results is given respectively for each specimen and sex in the form “number of heteroplasmic tissues showing transcription of M mtDNA / total number of heteroplasmic tissues”. Grey cells in the “Tissues” columns indicate heteroplasmic tissues. Empty cells in the “Tissues” columns indicate negative PCR results. Symbols used

*, positive amplification of M*orf* and/or *Mcox1* and/or M*cox2e*; -, sample not available; na, calculation not applicable.

## Discussion

### Mitochondrial heteroplasmy in freshwater mussels and other species with DUI

In the freshwater mussel *Unio crassus*, the presence of the M mtDNA (based on M*cox1* gene fragment amplification) was investigated in male and female somatic tissues but ascertained only in male soma and with various frequencies: 95% in the foot muscle (19/20), 70% in adductor muscle (17/20), 65% in mantle tissue (13/20), and 55% in gills tissue (11/20) [20]. In the present study, the M mtDNA has been found in male and also in female somatic tissues, as well as in male and female gonad tissues, in two freshwater mussel species, i.e., *Utterbackia peninsularis* and *Venustaconcha ellipsiformis* (amino acid divergences between F and M mtDNA-encoded proteins ~50% in both species). The tissue distribution patterns were variable, particularly in males, in which 86% of the foot samples contained the M mtDNA *versus* 70% for the adductor muscle, 64% for the mantle and 57% for the gills (in females 71% of the adductor muscles contained the M mtDNA *versus* 36% for the other somatic tissues). The M mtDNA was also found in four *U*. *peninsularis* female gonad samples (specifically, 4/5 in *U*. *peninsularis* and 0/5 in *V*. *ellipsiformis*).

Although our results on tissular distribution of M mtDNA in females are inconsistent with findings on *U*. *crassus* [20], they are in line with those observed in *Mytilus* spp. and *Ruditapes philippinarum*, i.e., (1) a more erratic presence of the M genome in female somatic tissues, which is consistent with the random segregation of sperm mitochondria in the early blastomeres of female embryos (and the failure to eliminate them), and (2) the nearly universal presence of it in male somatic tissues, which could be explained by the leakage of sperm mtDNA that escaped from the mitochondrial aggregate in male embryos (see [6] for a review). The detection of the M mtDNA in *U*. *peninsularis* female gonad samples is also in accordance with the presence of M mtDNA in *Mytilus* spp. eggs [28–30]. This suggests that, as in *Mytilus* spp. [29], the presence of M mtDNA in *U*. *peninsularis* eggs could lead, upon fertilization, to triplasmic individuals that carry an M genome inherited through the spermatozoon, as well as an F genome and an additional M inherited through the egg, but this remains to be demonstrated.

The non-detection of M mtDNA in *U*. *crassus* females [20], could be due to the rarity of M mtDNA in female tissues of that species and/or the limit of detection of the PCR assay (e.g., [15,16]). There are no quantitative data on M mtDNA copy number in freshwater mussels; only presence/absence has been tested by PCR so far ([20]; present study). It is possible that a higher quantity of M mtDNA in *U*. *peninsularis* and *V*. *ellipsiformis* female somatic tissues, in comparison to *U*. *crassus*, explains the detection of the M genome in the former two species but not in the latter. It is also possible that the presence of M mtDNA is somatic tissues depends on certain biological features specific to each species with DUI, leading to the observed variation.

### M-specific mitochondrial transcription in species with DUI

At the transcriptional level, we found that the M mtDNA is transcribed in male gonads, and in male and female soma of both *U*. *peninsularis* and *V*. *ellipsiformis*, but not in female gonads. Excluding male gonads, we found that 77% of male heteroplasmic tissues (60% in *U*. *peninsularis* and 89.5% in *V*. *ellipsiformis*) and 10% of female heteroplasmic tissues (6.7% in *U*. *peninsularis* and 20% in *V*. *ellipsiformis*) expressed the M genome. These results are consistent with those obtained for the marine mussel *M*. *edulis*, in which both male and female somatic tissues were found to express the M mtDNA [15]. They are also consistent with the results obtained for the marine clam *R*. *philippinarum*, in which male soma co-expresses both F and M mtDNAs [16]. However, our results are only partly consistent with protein-based studies showing the presence of the M mtDNA-encoded protein M*COX2* in male somatic and gonadal tissues, and in female gonads, but not in female somatic tissues of the freshwater mussel *V*. *ellipsiformis* [21,22]. In the current study, the M mtDNA has been found to be present and expressed in some female somatic tissues in both *V*. *ellipsiformis* and *U*. *peninsularis*, but not in female gonads. It is possible that the M mtDNA is also present and expressed in female gonads but below the limit of detection by our PCR and RT-PCR assays. Likewise, this could explain why some female gonad samples tested positive for the presence of M genome by PCR but negative for M genome expression by RT-PCR. Lastly, as suggested by Milani et al. [16], the presence of transcription may indicate a functional role of M mtDNA in DUI species, but transcriptional activity does not necessarily imply subsequent translation (and post-translational silencing might also exist). In our case, this could explain why transcription of the M genome was confirmed (present study) but no M mtDNA-encoded protein product was detected in the female soma of *V*. *ellipsiformis* ([21]).

Being the only mtDNA type in sperm [6,14,31], three general expressional patterns are theoretically possible for the M genome in species with DUI: (1) the first expression pattern would occur if the M mtDNA is expressed and functions only in the male gonads, specifically in spermatogenetic cells (as proposed by Obata, Sano & Komaru [19]); (2) the second expression pattern would occur if the M mtDNA is expressed in the male gonad and some or all male somatic tissues, but not in females; and (3) the other possible scenario is that the M mtDNA is expressed in the male and sometimes in the female gonads and some or all male and female somatic tissues (e.g., [18]; present study). We speculate that this last scenario is most probably the one that occurs in all species with DUI because, even if it remains to be demonstrated in *R*. *philippinarum*, this is the scenario that occurs in both Unionoida and Mytiloida. In this last group, it was perhaps more obvious in *M*. *edulis* [15], but although M mtDNA expression could not be observed in any of the male or female somatic tissues using the *in situ* hybridization approach in *M*. *galloprovincialis*, low levels of M mtDNA expression were detected in 35% of the female somatic tissue samples and in 70% of the male somatic tissue samples using an RT-qPCR approach [19].

### Cellular strategies for dealing with DUI and heteroplasmy

Three consecutive “checkpoints” have been proposed as the main steps of the DUI mechanism [14,32]: checkpoint #1 takes place shortly after fertilization when sperm mitochondria enter the egg and are maintained as an aggregate in male embryos, whereas they disperse in females (this phenomenon has been studied in *Mytilus* and *Ruditapes* but not in unionids yet); checkpoint #2 concerns the disappearance of the M mtDNA only from females, following the dilution and/or degradation of sperm mitochondria; and checkpoint #3 implies the segregation of M mtDNA in the male germ line and the F mtDNA in the female one, becoming the dominant mtDNA in the gonad and the only mitochondrial line transmitted by sperms and eggs, respectively. From the above data, it appears that checkpoints #2 and #3 can sometimes fail in species with DUI, leading to intra-tissular heteroplasmy in both male and female individuals. There exist some species in which male tissues consistently contain more M than F mtDNA molecules, such as *R*. *philippinarum*, probably because of a looser aggregation of sperm mitochondria in male embryos [13,14], and there exist some situations, owing to the stochastic nature by which mitochondria are passed on during cell division, in which some tissues in male or female individuals in DUI species could receive higher proportions of the M genome than others (e.g., [15]; present study).

Due to the strict maternal inheritance of mtDNA in animals, the mitochondrial genome is theoretically predicted to accumulate mutations that are beneficial or neutral in females, but potentially harmful when expressed in males (i.e., the so-called “mother’s curse”; [33]). DUI is the only system that allows selection to act directly on the male mitochondrial genome because males do not represent an evolutionary dead-end for mtDNA [4]. Therefore, due to its paternal inheritance, the M mitochondrial genome could accumulate mutations that are beneficial or neutral in males but potentially harmful when present and expressed in females, i.e. a “father’s curse”. One could hypothesize that DUI species have adopted measures to deal with this situation and ensure good cellular functions. For example, a functional adaptation of the nuclear genome to deal with mitochondrial heteroplasmy could theoretically be achieved by: (1) existence of nuclear isoforms that are only expressed and interact with a specific mtDNA (e.g., [4]); (2) silencing or downregulating of one mitochondrial lineage in heteroplasmic cells to restore a sort of homoplasmy (e.g., [16]); (3) maintaining nuclear heterozygosity (and/or genomic imprinting), with expression of specific “maternal” nuclear loci with the F mitotype and “paternal” loci with the M mitotype (e.g., [34]); and (4) assuring a different metabolism and role of the two sex-linked mitochondrial lineages. At this moment, all these hypotheses remain to be tested, and such studies are underway in our laboratory. For example, if nuclear isoforms exist that are only expressed and interact with the M genome, are they only expressed in males? This could explain why heteroplasmic males are more common than females with heteroplasmic somatic or gonadal tissues in DUI species [6,14,16, present study], i.e. they are more “apt” to tolerate heteroplasmy.

In any case, the observation that the M mtDNA is expressed in male and female somatic and gonadal tissues remains intriguing considering the levels of divergence between M and F mtDNAs and the fact that mitochondrial heteroplasmy in non-DUI species can lead to a wide range of deleterious effects (e.g., [17,18]). Among the possibilities discussed above, M-derived mRNAs could be maintained in a silenced/repressed state and/or post-transcriptional or post-translational gene silencing mechanisms could be involved (e.g., [16,35]). Although possible, this is inconsistent with the observation of an M mtDNA-encoded protein in *V*. *ellipsiformis* female gonads [22]. However, it has to be considered that this study involved a male-specific extension of the *cox2* gene, which could be involved in a function other than energy production [22], and hence more likely to be tolerated in a heteroplasmic state. In addition, because heteroplasmy affects a tissue or an organ mostly when the deleterious variant surpasses a certain copy-number threshold [18,36,37], it is possible that the threshold level above which the deleterious effect of M mtDNA could no longer be complemented by the coexisting F mtDNA would never be reached in somatic tissues of DUI bivalves. This could explain why both M mtDNA copy number and transcripts are maintained at very low levels in *Mytilus* spp., but this is rather inconsistent with the observation that the M mtDNA is much more prominent in male somatic tissues, even more than the F mtDNA, in the marine clam *R*. *philippinarum* [6,14]. However, it should be noted that all the above studies, including the present one, involve distantly-related species, and as suggested by Milani et al. [16], they might possess different biological features to deal with heteroplasmy.

## Conclusions

In this study we showed that F and M mitochondrial genomes are co-expressed in somatic tissues of two freshwater mussel species with doubly uniparental inheritance of mtDNA, i.e. *Utterbackia peninsularis* and *Venustaconcha ellipsiformis*. We also observed that the presence and expression of M mtDNA appears more often in males than in females. Because this trend was also reported in the distantly-related marine mussels and clam, i.e., that the M mtDNA is always present and expressed in the male (and more rarely in the female) gonads and in some or all male and also sometimes in female somatic tissues, we speculate that this could be the “general scenario” for all DUI species. Because of the deleterious nature of heteroplasmy, we also suggest the existence of different mechanisms in DUI species to deal with this potentially harmful situation, such as the existence of nuclear isoforms to function with the M genome and/or silencing mechanisms for the M mtDNA at the transcriptional, post-transcriptional or post-translational levels in somatic tissues of both sexes (and in female gonads) or only in females (if nuclear isoforms exist that are only expressed in males). All these hypotheses will require additional research in distantly-related DUI species from the four major orders Mytiloida, Nuculanoida, Unionoida, and Veneroida, which may have different mechanisms of action to deal with heteroplasmy.

## Supporting information

S1 Fig Adult individual for *U*. *peninsularis* (total shell length 46mm) and *V*. *ellipsiformis* (total shell length 45mm) (photo credit Richard T. Bryant and Deb Rose). (PDF)
