# Supplementary figures and images for: The extremely divergent maternally- and paternally-transmitted mitochondrial genomes are co-expressed in somatic tissues of two freshwater mussel species with doubly uniparental inheritance of mtDNA
Source: PLoS One. 2017 Aug 17;12(8):e0183529. doi: 10.1371/journal.pone.0183529 (PMC5560648; doi:10.1371/journal.pone.0183529)

*Utterbackia peninsularis*

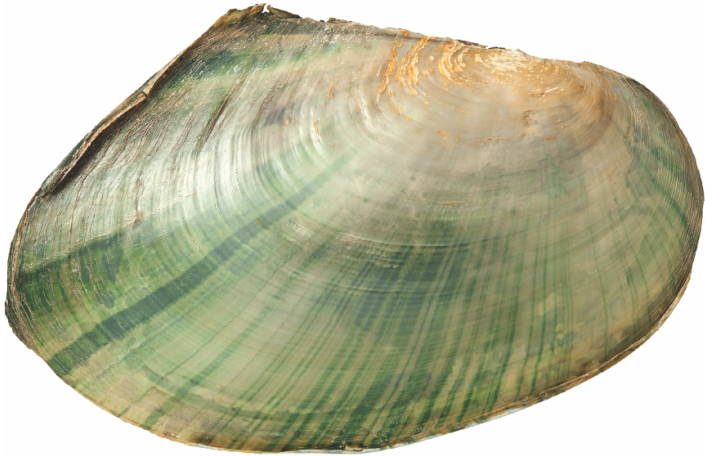

1 cm

*Venustaconcha ellipsiformis*

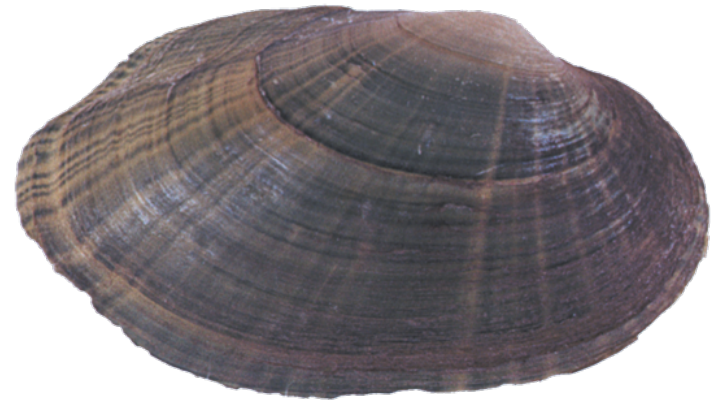

Supplement: S1 Fig — (PDF) [file pone.0183529.s001.pdf]
